# Cost of acute hospital treatment and initial aftercare for hospital-presenting self-harm in Ireland: national registry study

**DOI:** 10.1192/bjo.2026.10978

**Published:** 2026-03-02

**Authors:** Grace Cully, Brendan McElroy, Paul Corcoran, Beatriz Puertolas-Gracia, Eric Kelleher, Eugene Cassidy, Eve Griffin

**Affiliations:** National Suicide Research Foundation, Cork, Ireland; School of Economics, University College Cork, Cork, Ireland; School of Public Health, University College Cork, Cork, Ireland; CIBER Epidemiology and Public Health (CIBERESP), Madrid, Spain; Liaison Psychiatry Service, Cork University Hospital, Cork, Ireland; Department of Psychiatry and Neurobehavioural Science, University College Cork, Cork, Ireland

**Keywords:** Mental health services, self-harm, health economics, register-based study, emergency department

## Abstract

**Background:**

Understanding the economic cost of self-harm is essential for evaluating intervention cost-effectiveness and guiding funding allocation and service planning.

**Aims:**

To estimate the cost associated with self-harm presentations to hospital emergency departments and investigate key predictors of cost.

**Method:**

Data on presentations to hospital for self-harm in all Irish emergency departments were analysed for 2018 and 2019. Costs of hospital treatment following self-harm were identified (in 2019 euros) using top-down and bottom-up approaches. The perspective taken was that of the health service. Factors associated with costs were investigated using generalised linear models.

**Results:**

There were 25 053 self-harm presentations from 2018 to 2019. The average annual cost of self-harm was approximately €26.5 million; almost half of the total cost was due to repeat self-harm presentations (47.3%). The mean cost per presentation was €2117 (s.d. €1845), which incorporates acute hospital costs (mean €2067, s.d. €2127) and those of initial aftercare (mean €50, s.d. €69). Psychiatric and medical admissions were associated with highest costs, three times that of presentations resulting in emergency department discharge (incidence rate ratio (IRR) 3.01, 95% CI 2.72–3.36 and IRR 2.88, 95% CI 2.72–3.36, respectively). Other factors associated with higher costs included older age, emergency department medical assessment unit admission, receiving a psychosocial assessment and self-harm involving a firearm. Demographic and clinical predictors of cost varied according to care pathway.

**Conclusions:**

Significant costs associated with repeat attendances and hospital admission provide evidence for investment in emergency department services providing comprehensive care for those presenting with self-harm, as well as in community-based mental health services.

Hospital-presenting self-harm is a significant public health issue. Repetition of self-harm is common, with 16% of individuals who present with self-harm repeating within 12 months and 1 in 25 dying by suicide within 5 years.^1^ The healthcare costs associated with self-harm are considerable,^2–4^ and evidence suggests that these increase with repetition.^5^ Despite significant financial investment in programmes to prevent self-harm and suicide^6,7^ and calls for increased future investment globally,^8,9^ there is a dearth of high-quality costing studies of self-harm, with most focusing on general hospital costs without examination of aftercare costs,^2–4^ and just one study with national-level data.^10^ Prior Irish studies are limited to evidence on the economic costs associated with suicide.^11,12^ A comprehensive understanding of the economic costs of self-harm, including identifying drivers of high cost, is integral to the evaluation of the cost-effectiveness of preventative initiatives,^2,8^ and has the potential to play a pivotal role in both the allocation of funding and health policy development.^3,13^

Evidence on the cost of self-harm in Ireland is limited to a study of medical admission costs following acute mental health presentations to a paediatric emergency department.^14^ One of the most comprehensive calculations of the hospital costs of self-harm to date was conducted in England,^2^ where the costs of direct hospital medical care and psychosocial assessments were estimated for one hospital, reporting a mean cost per episode of self-harm of £809 (GBP).^2^ These costs were further extrapolated to develop an estimated annual cost of self-harm presentations across England of £129 million in 2013.^4^ The above analyses focused on immediate hospital costs only, without consideration of aftercare costs. Referrals to psychiatric out-patient care, specialist services and primary care are routine aftercare recommendations for individuals presenting to the emergency department following self-harm.

A Swiss study included out-patient appointments, in addition to medical and psychiatric in-patient costs, in its examination of the economic cost of self-harm in two hospitals in Basel, reporting that 97% of total costs were attributed to in-patient costs.^15^ However, only hospital-based out-patient appointments within 1 week of self-harm presentation were analysed, and no data were included on the costs of assessing patients. A recent study used national data to examine costs associated with hospital-presented self-harm in Norway (2012–2016),^10^ estimating a mean hospital cost per episode of self-harm of US$2248. This study included out-patient appointments within 1 month of the presentation in their cost estimation, but did not account for aftercare in the community or primary care. Furthermore, examination of predictors of cost was limited to treatment in hospital following admission from the emergency department. Factors associated with cost differences among those discharged home from the emergency department were not explored,^10^ which accounts for a substantial proportion of self-harm presentations.^16^ Although individual factors, including self-harm method, older age, suicidal intent, mental health diagnosis and socioeconomic deprivation, have been found to be determinants of cost, the main cost driver is the type of care provided.^2,15^

Using a national register of self-harm presentations, this study aimed to estimate the cost to health services associated with self-harm, including those related to ambulance use, emergency department consultation, psychosocial assessment, emergency department medical assessment unit admission, medical admission, psychiatric admission and all referrals to out-patient appointments, community mental health teams (CMHTs), addiction services or private counselling. The key predictors of self-harm presentation cost were also explored for all presentations, and separately for those that resulted in medical admission, psychiatric admission or discharge from the emergency department.

## Method

### Study setting and self-harm data

We undertook a retrospective analysis using data on all self-harm presentations to emergency departments in Ireland, to estimate the associated cost to health services. Data were obtained for the years 2018 and 2019 from National Self-Harm Registry Ireland (NSHRI).^17^ Self-harm is defined as ‘an act with non-fatal outcome in which an individual deliberately initiates a non-habitual behaviour, that without intervention from others will cause self-harm, or deliberately ingests a substance in excess of the prescribed or generally recognised therapeutic dosage, and which is aimed at realising changes that the person desires via the actual or expected physical consequences’.^17^

### Individual-level data

Core data items collected by NSHRI include: method(s) of self-harm, alcohol involvement, age, gender, date of attendance and medical card status. Method of self-harm was recorded according to ICD-10 codes for intentional injury (X60–84).^18^ Medical cards are means-test cards that facilitate free access to primary and public secondary healthcare and reduced payments for prescription medications.

Data relating to care pathways in the hospital were accessed, including whether the individual was brought to hospital by ambulance or emergency services, the provision of a psychosocial assessment in the emergency department, admission to a medical assessment unit in the emergency department and medical and psychiatric admission.^16,19^ For individuals discharged from the emergency department following treatment, data on recommended referrals were also recorded, including referral to a GP or out-patient appointment, CMHT, addiction services or private counselling. It was also recorded whether the patient self-discharged (i.e. left hospital without being seen, before a recommendation regarding next care could be made, or refused admission).

A first presentation by an individual was defined as a presentation to the emergency department for self-harm made during the study period (2018–2019) by individuals with no previous presentations. Self-harm history was acquired from the NSHRI data-set by examining all presentations made to emergency departments in the Republic of Ireland between 2007 and 2019. Repeat presentations were those made during the study period (2018–2019) by a person who had been recorded as presenting previously for self-harm. Recent self-harm history was categorised according to whether a person had presented for self-harm in the 12 months prior to their index presentation. Numbers of presentations per person during the study period were also calculated for each individual that presented in 2018 or 2019.

### Hospital-level data

Data at the level of the hospital at which a self-harm presentation occurred were also used. Publicly available information and data from published reports were used to generate the following hospital-level variables: type of hospital,^20^ psychiatric services onsite,^21^ availability of clinical nurse specialist^22^ and hospital location.

### Service utilisation and cost estimates

Costs of treatment following self-harm were estimated, in 2019 euros, from a variety of sources using both top-down and bottom-up approaches, in line with national guidelines for conducting cost analyses^23^ (Supplementary Table 1 available at https://doi.org/10.1192/bjo.2026.10978). The perspective taken was that of the health service provider. Two categories of direct healthcare costs were included: those of acute hospital care and those of initial aftercare.

### Acute hospital care

Published costings were used for arrival by ambulance or other emergency services,^24^ emergency department consultation unit cost and cost of psychiatric admission per bed-day.^25^ Costs of specific types of medical treatments or interventions within the emergency department are not costed individually, but are included in estimated emergency department consultation unit cost and the cost of admission to a medical assessment unit in the emergency department.

Unit cost of psychosocial assessment was estimated using the hourly wage for those who typically contribute to assessments (clinical nurse specialist (CNS), non-consultant hospital doctor and consultant psychiatrist) and an average length of assessment, estimated by clinical staff with appropriate experience. Salary estimates were derived from the Department of Health consolidated salary scales.^26^ In line with national guidelines,^23^ the median value from the salary scales was used to estimate the hourly wage and was adjusted for relevant costs, including overheads (25%). Full methodology on the costing of assessments is provided in Supplementary Tables 2 and 3. The cost of patients receiving a follow-up call from a CNS approximately 24 h post-discharge from the emergency department was based on CNS hourly wage, estimates of average call length and the proportion of assessed patients that receive a call.^7^

For all presentations in the NSHRI data-set that resulted in medical admission and/or admission to a medical assessment unit, cost of admission was predicted using data from the Hospital In-Patient Enquiry (HIPE) database. The HIPE database records all medical discharges, with all diagnoses and procedures coded according to ICD-10. The NSHRI data-set was then appended to the HIPE data-set. Costs were predicted using variables that were common to both data-sets: gender, age, method of self-harm and medical card status, using generalised linear models with gamma distribution and log link function. Further details on the methodology of costing of medical admission are provided in Supplementary Table 2.

Data on length of stay for emergency psychiatric admissions was not available for this study. A published average length for psychiatric admissions following hospital-presented self-harm was used in the cost estimate (7.3 days^27^). The impact of using this length of stay estimate was examined by conducting sensitivity analyses with estimated median and 25th and 75th percentile length of stay values (Supplementary Tables 4–6).

### Initial aftercare

Initial aftercare represents the recommended next point of care for the individual following emergency department discharge. Information on the time frame of aftercare and actual utilisation of services following discharge from the emergency department, i.e. whether the individual attended the follow-up appointment, was not available. Information on uptake of referrals was estimated based on a previous study that collected data on two subgroups of self-harm patients that presented to three Irish hospitals. Referral uptake data from this study have not been published, but the study procedures are described in published work.^28,29^ Based on referral data of 68 individuals between December 2014 and July 2019, the following rates of attendance at first appointment following discharge from the emergency department were costed: GP (91.7%), out-patient department (95.0%), community-based mental health team (87.0%), addiction services (50.0%) and private counselling (66.7%). The length of a first appointment with community-based mental health team, addiction services or private counselling was estimated to be 60 min by a liaison consultant psychiatrist. Published costings were used for out-patient^25^ and GP unit costs.^30^ The salary for each professional^23^ was used to estimate the unit cost of a first appointment with a CMHT, private psychological service, addiction service and 24 h follow-up telephone call (Supplementary Table 3).

Based on an audit by the National Clinical Programme for Self-harm and Suicide-related Ideation, it was estimated that 87% of assessed patients received a follow-up call; the average length of follow-up call was estimated to be 10 min by two CNSs with relevant experience. Again, labour costs and associated on-costs, as per national guidelines, were used to develop a unit cost for this call.

### Statistical analysis

Descriptive statistics, including measures of central tendency and distribution, were calculated for patient demographic and clinical characteristics, health service utilisation and costs. The predictors of health service costs, including a range of demographic, clinical and health service characteristics, were assessed using regression models. Models were run using the following dependent variables: total presentation cost, cost of presentations that resulted in medical admission, cost of presentations that resulted in psychiatric admission and cost of presentations that resulted in discharge from the emergency department. Predictors that were associated with the dependent variables at a significance level of *P* = 0.2 in crude models were included in the relevant adjusted models. Because the distribution of cost data is characterised by a positive skew and a long right tail (Supplementary Figs 1 and 2), generalised linear regression models were applied using a gamma distribution.^31^ Separate models were run with an identity link and a log link. The Bayesian information criterion, Akaike information criterion and the log-likelihood of the Pregibon link test were similar for both models (Supplementary Table 7). The results of the models using a log link are presented in this paper. Analyses were conducted using SPSS 27 for Windows (IBM, New York, USA; https://www.ibm.com/products/spss) and Stata 17 for Windows (StatCorp, Texas, USA; https://www.stata.com/).

### Ethical standards

This study has been approved by the Clinical Research Ethics Committee of the Cork Teaching Hospitals (reference nos ECM 4 (h) 11/5/2021 and ECM 3 (h) 13/02/2024. Ethical approval for the National Self-Harm Registry Ireland has been granted by the National Research Ethics Committee of the Faculty of Public Health Medicine.

### Consent statement

NSHRI complies with the European Union General Data Protection Regulation and operates a waiver of consent, granted by the Irish Health Research Consent Declaration Committee.

## Results

### Demographics and profile of self-harm

There were 25 053 presentations to Irish hospitals following self-harm during the study period (12 588 in 2018, 12 465 in 2019), involving 21 753 individuals (Table 1). Almost half of the presentations were repeat presentations (*n* = 11 860, 47.3%; Table 2), meaning that a previous presentation had been made by the presenting individual in the past (2007–2019). The majority of individuals presented once during the study period (*n* = 18 153, 72.5%), with 12.8% (*n* = 3205) presenting twice and 14.8% presenting 3 or more times (*n* = 3695). There were marginally more presentations by females (*n* = 13 832, 55.2%; Table 1) and the median age was 29 years (interquartile range 22 years). Most presentations involved individuals who were household residents (*n* = 23 253, 92.8%), and more than half were medical card-holders (*n* = 11 520, 58.6%). The most common method of self-harm was intentional drug overdose (IDO) (*n* = 15 555, 62.1%), followed by self-cutting (*n* = 7360, 29.4%; Table 1). Alcohol was involved in 30.6% of self-harm presentations (*n* = 7678), with more than half (*n* = 14 056, 56.5%) brought to hospital by ambulance.

**Table 1:** Demographics and clinical characteristics of self-harm presentations (*N* = 25 053)

Variable	*n* (%)
All	25 053 (100.0)
2018	12 588 (50.2)
2019	12 465 (49.8)
Gender	
Male	11 221 (44.8)
Female	13 832 (55.2)
Median age in years (IQR)	29 (22)
Age group	
<15 years	1087 (4.3)
15–44 years	18 586 (74.2)
45–64 years	4702 (18.8)
65+ years	678 (2.7)
Medical card-holder^a^	11 520 (58.6)
Residence status	
Household resident	23 253 (92.8)
Hospital in-patient	127 (0.5)
Prisoner	67 (0.3)
No fixed abode recorded	1207 (4.8)
Method of self-harm (ICD-10 code)	
Intentional drug overdose (X60–4)	15 555 (62.1)
Self-poisoning (X66–9)	486 (1.9)
Attempted hanging (X70)	2101 (8.4)
Attempted drowning (X71)	920 (3.7)
Self-cutting (X78)	7360 (29.4)
Jumping from a height (X80)	514 (2.1)
Blunt object (X79)	386 (1.5)
Jumping or lying in front of moving object (X81)	275 (1.1)
Self-immolation (X76)	110 (0.4)
Crashing motor vehicle (X82)	104 (0.4)
Firearm (X72)	15 (0.1)
Alcohol involvement	7678 (30.6)

IQR, interquartile range.

a. Missing in 5389 (21.5%) cases.

**Table 2:** Costs per person according to number of self-harm presentations, 2018–2019

Variables	*n* (%)	Mean cost, € (s.d.)	Total cost, € (%)		*n* (%)
			2018	2019	2018–2019
History of self-harm (*N* = 25 053 presentations)					
First presentation	13 193 (52.7)	2162 (2183)	14 593 523 (55.0)	13 923 704 (53.0)	28 517 227 (53.8)
Repeat presentation	11 860 (47.3)	2068 (2063)	12 157 301 (45.0)	12 366 056 (47.0)	24 523 357 (46.2)
Number of presentations per person, 2018–2019 (*N* = 21 753 persons)					
One presentation	18 153 (72.5)	2173 (2224)	2186 (2275)	2147 (2115)	32 487 715 (61.3)
Two presentations	3205 (12.8)	4271 (3251)	4186 (3104)	3345 (3126)	8 627 763 (16.3)
Three or more presentations	3695 (14.8)	10 080 (10 461)	9258 (8682)	6229 (6802)	11 925 107 (22.5)

History of self-harm includes any previous self-harm presentations from 2007 to 2019.

### Care pathways following self-harm

It was recorded in 94.5% of cases whether a psychosocial assessment was conducted in the emergency department. Of these, 17 561 (70.1%) received an assessment as part of their visit (Table 2). Approximately a quarter of self-harm presentations were admitted to a medical assessment unit in the emergency department (*n* = 16 925). Considering all presentations, 13.6% (*n* = 3406) self-discharged from the emergency department either before their treatment could be completed or against medical advice. More than a third were admitted to hospital for either medical treatment (*n* = 6074, 24.2%) or psychiatric in-patient care (*n* = 2735, 10.9%).

The remaining presentations (*n* = 12 838, 51.2%) were discharged from the emergency department following treatment. Half of those discharged from the emergency department were given a mental health-related referral (*n* = 6561, 51.1%), either an out-patient appointment (*n* = 4412, 34.3%), a referral to a community-based mental health team (*n* = 1404, 10.1%) or a referral to other psychological (*n* = 380, 2.9%) or addiction services (*n* = 365, 2.8%). A referral to attend a GP appointment was made for 9.6% of all presentations (*n* = 2407).

### Cost of hospital-presented self-harm

The total estimated cost of hospital-presented self-harm over the study period was €53 040 584: €26 750 824 in 2018 and €26 289 760 in 2019. The mean cost per presentation was estimated at €2117 (s.d. €1845), including both acute hospital costs (mean €2067, s.d. €2127) and those of initial aftercare (mean €50, s.d. €69). Cost per presentation ranged from €298 to €57 762, with a median of €1698. Medical admission was the care component with the highest estimated cost, accounting for 38.5% of the total cost, followed by admission to an emergency department assessment unit (22.1%) and psychiatric admission (17.1%) (Table 3).

**Table 3:** Costs associated with utilisation of hospital resources and recommended care pathways following hospital-presenting self-harm (2018–2019)

Variables	*n* (%)	Unit cost, €	Total cost per variable, € (% of total estimated cost)		
			2018	2019	2018–2019
Acute hospital care (*n* = 25 053)					
Brought by ambulance or emergency services	14 056 (56.5)	97	685 191 (2.6)	679 366 (2.6)	1 364 557 (2.6)
Emergency department consultation	25 053 (100)	298	3 751 224 (14.0)	3 714 570 (14.1)	7 465 794 (14.1)
Received psychosocial assessment^a^	17 561 (70.1)	102	892 695 (3.3)	901 584 (3.4)	1 794 278 (3.4)
Admitted to emergency department assessment unit^b^ (unit cost is mean cost per admission)	16 925 (25.8)	947	5 864 718 (21.9)	5 839 963 (22.2)	11 704 681 (22.1)
Admitted to medical bed (unit cost is mean cost per admission)	6074 (24.2)	3361	10 375 515 (38.8)	10 040 749 (38.2)	20 416 264 (38.5)
Admitted to psychiatric bed (unit cost is cost per bed-day)	2735 (10.9)	453	4 556 908 (17.0)	4 487 463 (17.1)	9 044 371 (17.1)
Estimated acute hospital care cost, € (mean (s.d.); total)		2067 (2127)	26 126 250	25 663,695	51 789 945
Initial follow-up for those discharged (*n* = 12 838)					
Discharged from emergency department without referral	2931 (11.7)	–	–	–	–
GP referral	2407 (9.6)	50	56 972 (0.2)	53 263 (0.2)	110 235 (0.2)
Out-patient appointment referral	4412 (17.6)	171	379 158 (1.4)	337 571 (1.4)	716 729 (1.4)
Community-based mental health team referral	1404 (5.6)	211	107 059 (0.4)	151 651 (0.6)	258 709 (0.5)
Psychological services referral	380 (1.5)	61	8710 (<0.1)	6902 (<0.1)	15 612 (<0.1)
Addiction services referral	365 (1.5)	61	8056 (<0.1)	11 417 (<0.1)	19 472 (<0.1)
24 h follow-up phone-call	15 102 (63.8)	9	64 619 (0.2)	65 262 (0.2)	129 881 (0.)
Estimated initial aftercare cost, € (mean (s.d.); total)		50 (69)	624 573	626 066	1 250 639
Total estimated cost (2018–2019), € (mean (s.d.); total)		2117 (2127)	26 750 824	26 289 760	53 040 584

GP, general practitioner.

13.6% (*n* = 3406) of patients either self-discharged before triage or before recommended care was provided, or refused to be admitted.

a Assessment unknown for 5.5% of presentations (*n* = 1371).

b Admission to emergency department observation unit unknown for 9.9% of presentations (*n* = 2491). Only presentations admitted to the emergency department that were not subsequently admitted to a medical ward are costed here (*n* = 12 364, 54.8%). Presentations that resulted in medical admission to a ward following emergency department admission are costed under medical admission.

Whereas the mean costs of first (€2162, s.d. €2183) and repeat presentations (€2068, s.d. €2063) were similar, almost half of the total cost of hospital-presented self-harm was due to repeat presentations (47.3%) (Table 2). Individuals who presented three or more times during the study period (14.8% of all individuals) had a mean cost of €10 080 (s.d. €10 461), accounting for 22.5% of the total cost.

### Predictors of cost

A multivariable generalised linear model was used to identify the main predictors of total presentation cost. Psychiatric admission and medical admission were the two factors associated with the highest total presentation cost, three times that of presentations that were discharged from the emergency department (IRR 3.01, 95% CI 2.72–3.36 and IRR 2.88, 95% CI 2.72–3.36, respectively). Admission to an emergency department medical assessment unit resulted in 1.8-fold higher cost compared with presentations discharged from the emergency department (95% CI 1.63–1.95). Those who received an assessment had higher costs compared with presentations where the patient was not assessed (IRR 1.78, 95% CI 1.63–1.95). Where psychosocial assessment and admission to the emergency department assessment unit were unknown, costs were significantly lower compared with presentations involving no assessment/admission (Table 4). Arriving by ambulance was associated with 1.12-fold higher costs compared with self-presentation (95% CI 1.09–1.14).

**Table 4:** Associations between cost and demographic, clinical and health service characteristics for all presentations, and for presentations resulting in medical admission, psychiatric admission and discharge from the emergency department

Variables	Model 1, total cost, € (*n* = 24 876)		Model 2, medical admission (*n* = 6039)		Model 3, psychiatric admission (*n* = 2711)		Model 4, discharged from emergency department (*n* = 12 735)	
	*b* (95% CI)	*P*-value	*b* (95% CI)	*P*-value	*b* (95% CI)	*P*-value	*b* (95% CI)	*P*-value
Female gender	1.03 (1.01 to 1.04)	<0.001	0.95 (0.94 to 0.96)	<0.001	1.01 (1.00 to 1.02)	0.062	1.07 (1.05 to 1.08)	<0.001
Age group (ref., 15–44 years)								
<15 years	0.99 (0.92 to 1.06)	0.750	0.88 (0.84 to 0.92)	<0.001	0.98 (0.95 to 1.00)	0.078	1.07 (1.00 to 1.13)	0.036
45–64 years	1.22 (1.19 to 1.25)	<0.001	1.44 (1.42 to 1.46)	<0.001	1.02 (1.01 to 1.04)	0.002	1.18 (1.16 to 1.21)	<0.001
65+ years	1.64 (1.53 to 1.76)	<0.001	2.20 (2.12 to 2.29)	<0.001	1.01 (0.97 to 1.05)	0.564	1.30 (1.23 to 1.37)	<0.001
IDO (X60–4)	0.90 (0.87 to 0.93)	<0.001	0.59 (0.56 to 0.62)	<0.001	1.01 (0.99 to 1.02)	0.456	1.05 (1.03 to 1.07)	<0.001
Self-poisoning (X66–9)	1.10 (1.04 to 1.15)	<0.001	1.25 (1.20 to 1.30)	<0.001				
Attempted hanging (X70)	1.00 (0.91 to 1.09)	0.926	2.03 (1.97 to 2.10)	<0.001	0.97 (0.95 to 0.98)	<0.001	0.71 (0.68 to 0.75)	<0.001
Attempted drowning (X71)			0.74 (0.72 to 0.78)	<0.001				
Self-cutting (X78)	1.00 (0.97 to 1.02)	0.736					1.05 (1.03 to 1.08)	<0.001
Firearm (X73)	1.72 (1.08 to 2.71)	0.021	3.02 (2.19 to 4.30)	<0.001	0.98 (0.93 to 1.03)	0.418	0.58 (0.44 to 0.77)	<0.001
Jumping from a height (X81)	1.2 (0.98 to 1.47)	0.074	3.17 (3.00 to 3.36)	<0.001	0.96 (0.94 to 0.98)	0.001	0.69 (0.62 to 0.76)	<0.001
Jumping or lying in front of moving object (X81)	0.97 (0.82 to 1.14)	0.693	3.32 (2.79 to 3.62)	<0.008	0.95 (0.93 to 0.98)	<0.001	0.58 (0.54 to 0.63)	<0.001
Crashing motor vehicle (X82)	1.13 (0.94 to 1.37)	0.189	3.51 (3.27 to 3.77)	<0.001	0.95 (0.93 to 0.97)	<0.001	0.59 (0.54 to 0.64)	<0.001
Self-immolation (X76)	1.18 (0.87 to 1.61)	0.281	3.38 (3.21 to 3.56)	<0.001	0.96 (0.94 to 0.98)	<0.001	0.66 (0.58 to 0.76)	<0.001
Blunt object (X79)	0.80 (0.71 to 0.90)	<0.001	2.99 (2.711 to 2.92)	<0.001	0.96 (0.94 to 0.98)	<0.001	0.61 (0.56 to 0.66)	<0.001
Alcohol involved	0.99 (0.98 to 1.00)	0.096					1.00 (0.99 to 1.01)	0.630
Medical card-holder								
Yes	0.95 (0.94 to 0.97)	<0.001	0.99 (0.97 to 1.01)	0.206				
Unknown	0.89 (0.84 to 0.95)	<0.001	0.41 (0.40 to 0.42)	<0.001				
Residence status (ref., household resident)								
Hospital in-patient	1.07 (0.94 to 1.22)	0.318	1.15 (0.90 to 1.47)	0.248	1.01 (0.97 to 1.06)	0.567	0.90 (0.82 to 0.98)	0.017
Prisoner	1.1 (0.89 to 1.36)	0.366	1.02 (0.98 to 1.06)	0.362	–		0.96 (0.91 to 1.01)	0.098
No fixed abode recorded	0.96 (0.93 to 1.00)	0.040	1.01 (0.99 to 1.03)	0.478	1.00 (0.97 to 1.02)	0.714	0.96 (0.94 to 0.98)	<0.001
Other	0.92 (0.87 to 0.97)	0.003	0.95 (0.87 to 1.04)	0.261	0.96 (0.94 to 0.98)	0.001	0.94 (0.92 to 0.97)	<0.001
Presented between 09.00 and 17.00 h	1 (0.99 to 1.01)	0.754	1.02 (1.01 to 1.03)	<0.001	0.99 (0.98 to 1.00)	0.068		
Presented at the weekend					0.99 (0.99 to 1.00)	0.003	1.00 (0.99 to 1.00)	0.615
History of self-harm in previous 12 months	0.99 (0.97 to 1.01)	0.353					0.98 (0.97 to 0.99)	<0.001
Brought by ambulance or emergency services	1.12 (1.09 to 1.14)	<0.001	1.07 (1.06 to 1.09)	<0.001	1.05 (1.04 to 1.06)	<0.001	1.11 (1.07 to 1.14)	<0.001
Received psychosocial assessment								
Yes	1.08 (1.02 to 1.14)	0.007	1.03 (1.01 to 1.51)	0.003	1.01 (0.96 to 1.06)	0.809	1.06 (1.02 to 1.10)	0.004
Unknown	0.59 (0.54 to 0.64)	<0.001	1.02 (0.96 to 1.06)	0.537	0.95 (0.89 to 1.02)	0.172	0.79 (0.74 to 0.85)	<0.001
Admitted to emergency department medical assessment unit								
Yes	1.78 (1.63 to 1.95)	<0.001	1.00 (0.86 to 1.16)	0.681	1.06 (1.02 to 1.10)	0.001	3.19 (3.05 to 3.35)	<0.001
Unknown	0.86 (0.78 to 0.94)	0.001	1.01 (0.98 to 1.04)	0.592	1 (0.97 to 1.04)	0.809	0.95 (0.91 to 1.00)	0.038
Admission status (ref., discharged from emergency department)								
Medical admission	2.88 (2.55 to 3.26)	<0.001						
Psychiatric admission	3.02 (2.72 to 3.36)	<0.001						
Self-discharged	0.61 (0.58 to 0.65)	<0.001						
Hospital type (ref., general)								
Children’s hospital	1.00 (0.84 to 1.19)	0.997	0.87 (0.43 to 1.06)	<0.001	0.94 (0.89 to 1.00)	0.062		
Local injury unit	0.93 (0.90 to 0.96)	<0.001	1.00 (0.91 to 1.09)	0.963	0.96 (0.89 to 1.02)	0.203		
Tertiary	0.97 (0.89 to 1.05)	0.438	1.00 (0.98 to 1.01)	0.614	1.02 (0.97 to 1.06)	0.502		
Psychiatric in-patient facilities on-site	1.02 (0.95 to 1.08)	0.638						
Dedicated liaison psychiatry services on-site	1.03 (0.99 to 1.08)	0.118						
Hospital location (ref., other city)								
Dublin city	0.98 (0.91 to 1.05)	0.549	1.01 (1.00 to 1.03)	0.140				
Town	0.98 (0.87 to 1.10)	0.672	0.99 (0.96 to 1.02)	0.641				
Constant	790.43 (685.61 to 911.27)	<0.001	3738.97 (2764.98 to 5056.05)	<0.001	3648.07 (3472.42 to 3832.62)	<0.001	432.25 (410.93 to 454.68)	<0.001

IDO, intentional drug overdose.

All models are adjusted general linear regressions using a gamma distribution and log link function.

Older age was associated with increasing cost, with presentations of people aged 65 years and older costing 1.64 times more than for those involving people aged 15–44 years (95% CI 1.53–1.76) (Table 4). Female gender was associated with higher presentation cost compared with male gender, but this difference was small (IRR 1.03, 95% CI 1.01–1.04). Self-harm methods associated with higher costs included firearm (IRR 1.72, 95% CI 1.08–2.71) and self-poisoning with substances other than drugs (IRR 1.10, 95% CI 1.04–1.15). Self-harm presentations involving a blunt object and those involving IDO were associated with lower costs compared with all other presentations (IRR 0.80, 95% CI 0.71–0.9 and IRR 0.90, 95% CI 0.87–0.93, respectively). Possession of a medical card (IRR 0.95, 95% CI 0.94–0.97) and unknown medical card status were associated with lower cost presentations compared with those involving non-medical card-holders (IRR 0.89, 95% CI 0.84–0.95). Local injury units were associated with lower cost presentations compared with general hospitals (IRR 0.93, 95% CI 0.90–0.96) (Table 4).

Further regression models were estimated examining presentation cost according to next care (Table 4). The strongest predictor of cost for presentations discharged from the emergency department, and those resulting in psychiatric admission, was admission to a medical assessment unit. Arriving by ambulance was associated with higher cost for presentations for all three care pathways. Receiving a psychosocial assessment was also a predictor of higher-cost presentations for those discharged from the emergency department and those admitted medically.

Consistent with the overall findings, older age was one of the strongest predictors of high-cost presentations for medical admission, and for those discharged from the emergency department. However, for presentations that resulted in discharge from the emergency department, female gender was associated with significantly higher presentation cost compared with male, with female gender being a predictor of lower-cost presentations for those admitted medically. History of self-harm was associated with lower-cost presentations for those discharged from the emergency department, compared with presentations involving people presenting for the first time.

Self-harm method was a strong predictor of cost of medical admission, with significantly higher costs for most of the methods associated with high lethality, including crashing a motor vehicle, self-immolation, jumping or lying in front or a moving object, jumping from a height, firearm, attempted hanging and self-poisoning. For those admitted medically, self-harm with a blunt object was also associated with increased cost, with IDO and attempted drowning being associated with lower cost. For presentations that resulted in discharge from the emergency department, more lethal and less common methods of self-harm, including firearm, jumping or lying in front of a moving object, crashing a vehicle, blunt object, self-immolation, jumping from a height and attempted hanging, were predictors of lower cost, whereas IDO and self-cutting were associated with higher cost presentations. For presentations that resulted in psychiatric admission, although the associations between cost and methods were not as strong, jumping or lying in front of moving object, crashing a motor vehicle, self-immolation, blunt object, jumping from a height and attempted hanging were significant predictors of lower-cost presentations. Alcohol did not impact on presentation cost in adjusted models.

## Discussion

### Main findings

This study provides a national estimate of costs related to hospital-presented self-harm based on comprehensive data from a national registry of self-harm. The mean cost of self-harm was €2117 per presentation to hospital, with a total estimated annual cost of approximately €26.5 million. Almost half of the total cost of hospital-presented self-harm was due to repeat self-harm presentations. The strongest drivers of total cost were admission to a psychiatric or medical ward, admission to an emergency department assessment unit, receiving a psychosocial assessment, self-harm involving a firearm and older age. Demographic and clinical predictors of cost varied according to care pathway.

Few studies detailing the costs of hospital-presented self-harm are available, with just one previous study using national data.^15^ The current study advances the costing of self-harm conducted in previous studies in Norway^15^ and England^2^ by including ambulance costs and those of initial aftercare beyond the hospital setting. The mean cost of a self-harm presentations in Ireland was comparable to the Norwegian estimate, while the English study reported a substantially lower mean cost. This may be partly due to the addition of ambulance costs, a significant driver of high-cost presentations in the present study, and the inclusion of aftercare costs. Furthermore, our estimate of the cost of psychosocial assessments is more than twice that of assessment in the English study. Different approaches to estimating assessment cost were taken; the current study estimated the average cost of conducting one assessment and attributed this cost to each presentation for which an assessment was carried out, whereas Tsiachristas et al^2^ computed the full-time-equivalent cost of each member of hospital staff involved in conducting assessments and divided this total by the number of assessments. Future work in this area would benefit from collecting primary data on assessments in order to calculate a more accurate estimate. Our findings indicate that the type of hospital was associated with presentation cost. Thus, the inclusion of multiple hospitals in the Irish and Norwegian estimates (latter, *n* = 14), compared with the single centre from which the English estimate was derived, may also have contributed to the difference in the average cost of presentations.

The cost of initial aftercare was a small proportion of the overall cost in the present study, in line with findings from the studies in Switzerland^4^ and Norway.^15^ Providing a more comprehensive estimate of aftercare than previous research, the current study included appointments beyond hospital out-patient services, including mental health and addiction services within community and primary care, as well as costing the follow-up call made by emergency department-based clinical nurse specialists. However, there is a need for a better estimate of uptake of, and attendance at, community-based appointments. In addition, a majority of those discharged from a medical ward or psychiatric in-patient setting are likely to receive referral, which was not accounted for in this study. Therefore, reported costs are likely to be an underestimation of the costs related to hospital-presented self-harm, and more work is needed on mapping care pathways following self-harm to more comprehensively estimate the cost of aftercare.

Consistent with existing evidence,^2,10,15^ the type of care provided was the strongest driver of cost in the current study. Although receiving a psychosocial assessment and admission to medical assessment units were predictors of higher-cost presentations, psychiatric and medical admissions were associated with the highest cost presentations, accounting for more than half of the total annual cost. Admission has been shown to be largely driven by hospital-related factors that are independent of individual factors related to treatment needs.^19^ In line with mental health policy in Ireland,^32^ and internationally,^33^ efforts to reduce unnecessary admissions to hospital following self-harm should be prioritised. Evidence indicates that medical assessment units in the emergency department are associated with reduction in both admission rates and length of stay,^19,34^ and consequently with lower cost.^34^ The current findings may indicate the use of these units to avoid admissions, given that admission to a medical assessment unit was a strong driver of cost for presentations resulting in emergency department discharge, but were not predictors of cost for those medically admitted.

Differences in staffing of services providing care for those who present to the emergency department with self-harm could also have an impact on cost, due to differences in the availability of out-of-hours care.^35^ Investment in services within the emergency department that provide comprehensive assessment and care-planning for those who present with self-harm have also been shown to reduce admission rates, and to increase referrals to community-based aftercare^35,36^ and reduce hospital costs.^36,37^ The lower costs associated with initial aftercare compared with admission costs, reported in the present study, indicate that reducing admission with a corresponding increase in referrals would result in a reduction in costs to the health service overall. Acute referral is possible only with adequate resourcing of mental health and addiction services in the community. This study also highlights the significant cost to health services as a result of repeat self-harm presentations to the emergency department. Although the costs associated with first and repeat presentations were similar, almost half of the total cost of hospital-presented self-harm was due to repeat presentations. This finding is reflective of the high proportion of presentations that are due to repeat attendances, and is indicative of ongoing distress for many individuals who present to the emergency department with self-harm. Previous evidence highlights challenges in the availability and accessibility of mental health and addiction services to which individuals being discharged from the emergency department can be referred. Increasing the capacity of under-resourced community services providing interventions with the potential to reduce repeat self-harm should be prioritised to ensure that individuals are receiving the support they need, and to save future hospital costs. Evidence suggests that many individuals repeatedly presenting with self-harm have a personality disorder diagnosis and may benefit from specialised interventions in the community.^38^ Further investment in services for those with multiple self-harm presentations is warranted to help meet the needs of these individuals, and thereby to reduce the significant cost associated with repeat attendances.

Individual factors also emerged as predictors of higher cost. A linear association between older age and higher-cost presentations was identified, with lowest-cost presentations for those under the age of 15 years and highest for those aged 65 years and over. This association is particularly marked in the analysis of costs associated with medical admission. Research indicates that older people who attend hospital with self-harm frequently present with high suicidal intent, are often living alone and have chronic mental health conditions.^39^ These factors are indicative of the seriousness and complexity of self-harm presentations among older people. Furthermore, risk of suicide following self-harm is elevated among older people. In the emergency department setting, more extensive care is likely to be allocated to those at higher risk of adverse outcomes,^39^ which may help to explain some of the elevated cost associated with presentations in this group. Comorbid physical health complaints are a common risk factor for self-harm in older adults,^40^ which may have an impact on the medical care required when they present, and are admitted, to hospital following self-harm. Furthermore, the types of drugs involved in self-harm presentations by older people may also contribute to more extensive care, with high prescribing rates of tricyclic antidepressants reported in a large cohort study of older primary care patients in the UK,^40^ a drug type that is not recommended for prescription due to its toxicity.

Examining predictors of cost according to care pathways identified different patterns of association between demographic and clinical factors and presentation cost. For presentations that resulted in discharge from the emergency department, more lethal methods of self-harm were predictors of lower cost, with IDO and self-cutting associated with higher cost. Self-harm presentations to hospital range from low-severity acts requiring no medical intervention to high-severity acts requiring significant intervention to prevent a fatal outcome. The observed findings among those discharged home from the emergency department may reflect that presentations involving high-lethality methods can be of low severity, requiring no medical intervention, whereas IDO and self-cutting presentations may be more likely to require some level of medical treatment, even for minor injuries. By contrast, most methods associated with high lethality were predictors of higher cost for those medically admitted, with IDO and attempted drowning predicting lower costs. This is consistent with previous studies that have reported that methods of self-injury, particularly high-lethality methods, were associated with higher costs compared with IDO for admitted patients.^2,10^ These findings may indicate that more extensive medical treatment is needed for presentations involving these high-lethality methods when admission to a medical ward is needed. Indeed, Tsiachristas et al^2^ reported longer stays in hospital for those admitted following self-harm. For presentations that resulted in discharge from the emergency department, female gender was associated with higher presentation cost compared with male, and was a predictor of lower-cost presentations for those admitted medically. Males generally present with higher-severity self-harm presentations compared with females,^41,42^ and thus males admitted medically may require more extensive intervention, such treatment in an intensive care unit, which is one of the highest-cost types of care.^10^ Information on the specific treatments received across the different care pathways is needed to develop a full understanding of the observed differences.

### Strengths and limitations

In addition to costing immediate care provided within the hospital, this study developed cost estimates of the next step in care following discharge from the emergency department, providing a more comprehensive estimate than previous studies in this area of the cost related to a presentation to the emergency department as a result of self-harm. The large national data-set with standard operating procedures for data collection is a further strength of this study, allowing for stratification of the data according to care pathway and the use of advanced modelling techniques to provide robust estimates of costs. Notably, previous national studies have extrapolated findings from one hospital to derive a national estimate,^4^ or used national self-harm data that were not collected in a standardised way.^10^ However, there are some limitations to this study. Costs solely relate to direct healthcare costs and do not capture indirect costs, such as loss of productivity or costs to the patient and their family members or carers. Duration of, and personnel conducting, psychosocial assessments and aftercare appointments were estimated based on expert opinion. Due to a lack of applicable published evidence, attendance at aftercare appointments was estimated from unpublished data collected from 2014 to 2019, including from a small sample of individuals (*N* = 68). Accurate data in these areas would improve the precision of cost estimates. In addition, we did not have information on specific treatments received, such as administration of antidotes for IDOs and admission to intensive care, or information on mental health conditions and comorbid alcohol or substance use, which may be important predictors of cost. Furthermore, the regression model used to predict costs associated with medical admission was limited to four variables, and did not include potentially important predictors of cost such as length of stay or treatment received. This is likely to have impacted the accuracy of our medical admission cost estimate. Our estimation of cost associated with psychiatric admission was based on a published average length of stay, which may have impacted the precision of our cost estimates. However, the results of sensitivity analyses, with costs that were developed based on alternative lengths of stay, are consistent with the findings presented in the paper. Whether or not an individual had a history of presenting with self-harm was determined based on whether they presented to hospital between 2007 and 2019; individuals who presented before 2007 and did not re-present until the study period (2018–2019) may have been misclassified as having no self-harm history.

This study highlights the substantial costs associated with hospital-presented self-harm, almost half of which is due to repeated self-harm. The significant costs associated with repeat attendances and admission to hospital following self-harm provide evidence to support the investment in services within the emergency department that provide comprehensive assessment and care-planning for those who present with self-harm. Community-based psychosocial service interventions that target populations to prevent re-presentation, including those for individuals with personality disorder and addiction, should be prioritised. In parallel, high-impact service user interventions across emergency departments and community settings need to be developed, resourced and delivered for those individuals with multiple self-harm repeat presentations.

## Supporting information

Cully et al. supplementary material Cully et al. supplementary material

## Data Availability

Access to data from NSHRI may be requested by contacting the National Suicide Research Foundation (info@nsrf.ie).

## Supplementary material

The supplementary material is available online at https://doi.org/10.1192/bjo.2026.10978
