# Supplementary material for: Cost of acute hospital treatment and initial aftercare for hospital-presenting self-harm in Ireland: national registry study
Source: BJPsych Open. 2026 Mar 2;12(2):e78. doi: 10.1192/bjo.2026.10978 (PMC12963842; doi:10.1192/bjo.2026.10978)
Supplement: Cully et al. supplementary material [file S2056472426109788sup001.docx]

**Supplementary material - Cost of acute hospital treatment and initial aftercare for hospital-presenting self-harm in Ireland: A national registry study**

**Supplementary Table 1. Overview of the development of cost estimates for each care item.**

| Resource | Proportion of SH presentations | Uptake | Duration | Detailed unit cost (€) | Total unit cost | Unit cost with uptake | Total cost | Sources |
| --- | --- | --- | --- | --- | --- | --- | --- | --- |
| Ambulance costs | 14,056 (56.5) | na | na | 97.08 per presentation | €97 | €97 | €1,364,556 | Murphy et al., 2020 (5) |
| Emergency department consultation | 25053 (100) | na | na | 298 per presentation | €298 | €298 | €7,465,794 | Cost per presentation: Table 5.1, Keegan et al., 2020 (6) |
| Psychosocial assessment | 17,561 (70.1) | na | 75 minutes | - 0.86 per minute for CNS (54.78% assessments) - 0.93 per minute for junior doctor (45.22% assessments) - 3.53 per minute for consultant psychiatrist | €102 | €102 | €1,794,278 | LOA: clinical estimate.  Cost of personnel: Supplementary Table 3.  Personnel conducting assessments: Pitman et al. CNS (54.78%) and NCHD (45.22%). Clinical estimate: add 10 minutes consultant time per assessment. |
| Admitted to ED assessment unit | 16,925 (25.8) | na | na |  | €766^a^ | €766^a^ | €9,444,392 | Hospital In-Patient Enquiry (HIPE) dataset (7) |
| Admitted to medical bed | 6,074 (24.2) | na | na |  | €3,271 | €3,271 | €17,269,017 | Hospital In-Patient Enquiry (HIPE) dataset (7) |
| Admitted to psychiatric bed | 1,638 (6.5) | na | 7.3 days | 453 per bed day | €453 | €3,307 | €5,416,702 | Duration: Quinlivan et al., 2019;  Cost per bed day: p 75, Keegan et al., 2020 (ESRI report) |
| Transferred to another psychiatric hospital | 1,097 (4.4) | na | 7.3 days | 453 per bed day | €453 | €3,307 | €3,627,669 | Duration: Quinlivan et al., 2019 (8) Cost per bed day: p 75, Keegan et al., 2020 (6) |
| Discharged from emergency department | 12,493 (49.9) | - | - | - | - | - |  |  |
| Discharged without referral | 2,931 (11.7) | - | - | - | - | - |  |  |
| General practitioner referral | 2,407 (9.6) | 92% | na | 49.78 per appointment | €50 | €46 | €110,235 | Smith et al., 2021 (9) |
| Outpatient appointment referral | 4,412 (17.6) | 95% | na | 171 per appointment | €171 | €162 | €716,729 | Duration: unpublished data from IMPRESS study (10,11) Cost per appointment: Table 5.1, Keegan et al., 2020 (6) |
| Community-based mental health team referral | 1,404 (5.6) | 87% | 60 minutes | 211.80 per hour | €211 | €184 | €258,709 | Duration: unpublished data from IMPRESS study (10,11) Length of appointment: Clinical estimate  Cost of personnel: Supplementary Table 3 (below) |
| Psychological services referral | 380 (1.5) | 67% | 60 minutes | 61.32 per hour | €61 | €41 | €15,612 | Duration: unpublished data from IMPRESS study (10,11) Cost of personnel: Supplementary Table 3 (below) |
| Addiction services referral | 365 (1.5) | 50% | 60 minutes | 61.32 per hour | €61 | €53 | €19,472 | Uptake: unpublished data from IMPRESS study (10,11) Cost of personnel: Supplementary Table 3 (below) |
| Crisis nurse phone-call | 15,102 (63.8)^3^ | 86% * | 10 minutes | 51.88 per hour (0.86 per minute) | €9 | €9 | €129,881 | Uptake: NCPSHI model of care, 2022 (12)  Length of appointment: Clinical estimate Cost of personnel: Supplementary Table 3 (below) |

CNS=clinical nurse specialist. NCHD=non-consultant hospital doctor. *Percentage of assessed presentations.

**Supplementary Table 2. Detailed methodology of costing psychosocial assessment and medical admission**

| ***Psychosocial assessment*** | The type of personnel conducting the assessment was estimated from published research (1): 54.8% conducted by a CNS and 45.2% by a non-consultant hospital doctor**.** Ten minutes of consultant input per assessment was also incorporated into the cost estimate. The average length of time spent with the patient conducting the face-to-face portion of the assessment was independently estimated by four clinical nurse specialists (CNSs) with relevant experience and the mean length was calculated (75 minutes). This estimate was considered accurate by a liaison consultant psychiatrist. The hourly wage for CNSs and NCHDs were used to estimate the unit cost of a biopsychosocial assessment. Salary estimates were derived from the Department of health consolidated salary scales (2). In line with national guidelines (3), the median value from the salary scales was used to estimate the hourly wage and was adjusted for relevant costs including overheads (25%) (Supplementary table 2). |
| --- | --- |
| ***Medical admission*** | Data were obtained from the Hospital In-Patient Enquiry (HIPE) database, which records all medical discharges, and all diagnoses and procedures are coded according to ICD-10 (4). Data on all emergency medical admissions in 2018 and 2019 including an ICD-code relating to self-harm (X60-X84) were obtained from this database including details on age, sex, medical card status, method of self-harm and length of stay, type of admission (admission to ED medical assessment unit and/or medical ward), and cost for each presentation. The Registry dataset was then appended to the HIPE dataset. Two generalised linear models were used to predict costs of admission to an ED assessment unit and a medical ward from the HIPE dataset to the Registry dataset. The following variables, common to both datasets, were used in the regression models: sex, age, method of self-harm, medical card status. We used gamma distribution for the models due to the skewed nature of the data, with many low-cost admissions and fewer admissions associated with a very high cost. We used the log link function to avoid cost predictions below zero. As a sensitivity analysis, we predicted costs onto the HIPE dataset also, to compare these with the actual costs, calculating residuals and error values and comparing the range and central tendencies. This analysis indicated that the cost predictions were within the range of actual costs, with a similar mean cost (predicted mean €4023; SD 3773 vs actual mean €4012; SD 14,444). There was an overprediction of the lowest costs and an underprediction of the highest costs due to some very high outliers in the actual costs. |

**Supplementary Table 3. Health staff pay and related costs.**

| Staff Type | Basic Pay | PRSI & Pensions ^2^ | Overheads^3^ | Total Cost | Hourly Rate ^4^ | Rate Per Minute |
| --- | --- | --- | --- | --- | --- | --- |
| Clinical Psychologist^1^ | 69,359.00 | 0,230.45 | 17,339.75 | 96,929.20 | 64.87 | 1.08 |
| Clinical Nurse Specialist | 55,469 | 8,181.68 | 13,867.25 | 77,517.93 | 51.88 | 0.86 |
| Junior doctor^5^ | 59,609 | 8,792.33 | 14,902.25 | 83,303.58 | 55.75 | 0.93 |
| Consultant Psychiatrist | 226,461 | 33,403.00 | 56,615.25 | 316,479.25 | 211.80 | 3.53 |
| Counsellor / therapist^6^ | 65,565 | 9,670.84 | 16,391.25 | 91,627.09 | 61.32 | 1.02 |

Notes

1. Mid-point of Clinical Psychologist salary scale. Source: Department of Health consolidated salary scales Feb 2022 - Jul 2022 (13).
2. Employer’s contributions to PRSI (4%) and Pension (10.75%) of basic pay. Based on HIQA guidelines for the economic evaluation of health technologies, appendix 4 (14).
3. 25% of basic pay. Based on HIQA guidelines for the economic evaluation of health technologies, appendix 4 (14).
4. Total annual pay costs divided by (43 weeks*34.75 hours pw). 43 weeks is 52 weeks less 9 weeks leave (2 weeks public holidays, 5 weeks annual leave, 2 weeks sick leave). Based on HIQA guidelines for the economic evaluation of health technologies, appendix 4 (14).
5. Mid-point NCHD - intern, senior house officer and registrar
6. Mid-point of counsellor therapist and counsellor therapist, senior. Based on assumption, informed by methods used by Personal Social Services Research Unit in UK (15)

**Supplementary Table 4. Calculation of psychiatric admission cost with alternative length of stay estimates.**

| LOS | Resource | Proportion of SH presentations | Total unit cost | Unit cost with uptake | Total cost | Psychiatric admission total cost ^1^ | Mean presentation cost (SD) ^2^ | Sources |
| --- | --- | --- | --- | --- | --- | --- | --- | --- |
| 7.35 days (mean) | Admitted to psychiatric bed | 1,638 (6.5) | €453 | €3,307 | €5,416,702 | **€9,044,371** | **€2,117 (2,127)** | Duration: Quinlivan et al., 2019 (8) Cost per bed day: p 75, Keegan et al., 2020 (6) |
|  | Transferred to another psychiatric hospital | 1,097 (4.4) | €453 | €3,307 | €3,627,669 |  |  |  |
| 4.5 days (estimated 25^th^ percentile) | Admitted to psychiatric bed | 1,638 (6.5) | €453 | €2,039 | €3,339,063 | **€5,575,298** | **€1,979 (2,044)** | Duration: Quinlivan et al., 2019 ^3^;  Cost per bed day: p 75 (6) |
|  | Transferred to another psychiatric hospital | 1,097 (4.4) | €453 | €2,039 | €2,236,235 |  |  |  |
| 6.6 days (estimated median) | Admitted to psychiatric bed | 1,638 (6.5) | €453 | €2,990 | €4,897,292 | **€8,177,103** | **€2,083 (2,100)** | Duration: Quinlivan et al., 2019 ^3^;  Cost per bed day: p 75, Keegan et al., 2020 (6) |
|  | Transferred to another psychiatric hospital | 1,097 (4.4) | €453 | €2,990 | €3,279,811 |  |  |  |
| 9.1 days (estimated 75^th^ percentile) | Admitted to psychiatric bed | 1,638 (6.5) | €453 | €4,122 | €6,752,327 | **€11,274,161** | **€2,206 (2,216)** | Duration: Quinlivan et al., 2019 (8) ^3^ Cost per bed day: p 75, Keegan et al., 2020 (6) |
|  | Transferred to another psychiatric hospital | 1,097 (4.4) | €453 | €4,122 | €4,521,834 |  |  |  |

Notes

1. Psychiatric admission total cost includes admission to psychiatric bed and transfer to another psychiatric hospital.
2. Mean presentation cost refers to all presentations, i.e. it is not limited to those that resulted in psychiatric admission.
3. Healthcare costs typically follow a Gamma distribution. Given this distribution, and a mean of 7.3 days, we estimated plausible values of a 25th percentile of 4.5 nights, a median of 6.6 nights and a 75% percentile of 9.1 nights.

**Supplementary Table 5. Associations between cost and demographic, clinical and health service characteristics for all presentations (Model 1; n=24,876) assuming length of stay in psychiatric setting of 7.3 days (mean), 4.5 days (estimated 25^th^ percentile), 6.1 days (estimated median), and 9.1 days (estimated 75^th^ percentile).**

|  | **LOS: 7.3 days** | | **LOS: 4.5 days** | | **LOS: 6.1 days** | | **LOS: 9.1 days** | |
| --- | --- | --- | --- | --- | --- | --- | --- | --- |
|  | *b* (95% CI); p value | | *b* (95% CI); p value | | *b* (95% CI); p value | | *b* (95% CI); p value | |
| **Female** | 1.03 (1.01 to 1.04) | <0.001 | 1.03 (1.01 to 1.04) | <0.001 | 1.03 (1.01 to 1.04) | <0.001 | 1.03 (1.01 to 1.04) | <0.001 |
| **Age group**  (ref, 15-44 years) |  |  |  |  |  |  |  |  |
| <15 years | 0.99 (0.92 to 1.06) | 0.750 | 0.99 (0.92 to 1.06) | 0.778 | 0.99 (0.92 to 1.06) | 0.755 | 0.99 (0.92 to 1.06) | 0.74 |
| 45-64 years | 1.22 (1.19 to 1.25) | <0.001 | 1.22 (1.20 to 1.25) | <0.001 | 1.22 (1.19 to 1.25) | <0.001 | 1.22 (1.19 to 1.25) | <0.001 |
| 65+ years | 1.64 (1.53 to 1.76) | <0.001 | 1.64 (1.53 to 1.76) | <0.001 | 1.64 (1.53 to 1.76) | <0.001 | 1.64 (1.53 to 1.76) | <0.001 |
| **IDO (X60-X64)** | 0.90 (0.87 to 0.93) | <0.001 | 0.90 (0.87 to 0.93) | <0.001 | 0.90 (0.87 to 0.93) | <0.001 | 0.90 (0.87 to 0.93) | <0.001 |
| **Self-poisoning (X66-69)** | 1.10 (1.04 to 1.15) | <0.001 | 1.10 (1.04 to 1.15) | 0.001 | 1.10 (1.04 to 1.15) | <0.001 | 1.10 (1.04 to 1.15) | <0.001 |
| **Attempted hanging (X70)** | 1.00 (0.91 to 1.09) | 0.926 | 0.99 (0.91 to 1.09) | 0.889 | 1.00 (0.91 to 1.09) | 0.919 | 1.00 (0.91 to 1.09) | 0.94 |
| **Self-cutting (X78)** | 1.00 (0.97 to 1.02) | 0.736 | 1.00 (0.97 to 1.02) | 0.763 | 1.00 (0.97 to 1.02) | 0.741 | 1.00 (0.97 to 1.02) | 0.726 |
| **Firearm (X73)** | 1.72 (1.08 to 2.71) | 0.021 | 1.71 (1.09 to 2.71) | 0.021 | 1.72 (1.09 to 2.71) | 0.021 | 1.72 (1.08 to 2.72) | 0.021 |
| **Jumping from a height (X81)** | 1.2 (0.98 to 1.47) | 0.074 | 1.20 (0.98 to 1.47) | 0.079 | 1.20 (0.98 to 1.47) | 0.075 | 1.20 (0.98 to 1.47) | 0.072 |
| **Jumping or lying in front of moving object (X81)** | 0.97 (0.82 to 1.14) | 0.693 | 0.97 (0.82 to 1.14) | 0.669 | 0.97 (0.82 to 1.14) | 0.689 | 0.97 (0.83 to 1.14) | 0.702 |
| **Crashing motor vehicle (X82)** | 1.13 (0.94 to 1.37) | 0.189 | 1.13 (0.94 to 1.36) | 0.201 | 1.13 (0.94 to 1.37) | 0.191 | 1.14 (0.94 to 1.37) | 0.185 |
| **Self-immolation (X76)** | 1.18 (0.87 to 1.61) | 0.281 | 1.18 (0.87 to 1.61) | 0.284 | 1.18 (0.87 to 1.61) | 0.281 | 1.18 (0.87 to 1.61) | 0.279 |
| **Blunt object (X79)** | 0.80 (0.71 to 0.90) | <0.001 | 0.80 (0.70 to 0.90) | <0.001 | 0.80 (0.70 to 0.90) | <0.001 | 0.80 (0.71 to 0.90) | <0.001 |
| **Alcohol involved** | 0.99 (0.98 to 1.00) | 0.096 | 0.99 (0.98 to 1.00) | 0.079 | 0.99 (0.98 to 1.00) | 0.092 | 0.99 (0.98 to 1.00) | 0.103 |
| **Medical card holder** |  |  |  |  |  |  |  |  |
| Yes | 0.95 (0.94 to 0.97) | <0.001 | 0.95 (0.94 to 0.97) | <0.001 | 0.95 (0.94 to 0.97) | <0.001 | 0.95 (0.94 to 0.97) | <0.001 |
| Uknown | 0.89 (0.84 to 0.95) | <0.001 | 0.89 (0.84 to 0.95) | <0.001 | 0.89 (0.84 to 0.95) | <0.001 | 0.89 (0.84 to 0.95) | <0.001 |
| **Residence status**  (ref, household resident) |  |  |  |  |  |  |  |  |
| Hospital in-patient | 1.07 (0.94 to 1.22) | 0.318 | 1.07 (0.94 to 1.22) | 0.301 | 1.07 (0.94 to 1.22) | 0.315 | 1.07 (0.94 to 1.22) | 0.325 |
| Prisoner | 1.1 (0.89 to 1.36) | 0.366 | 1.10 (0.89 to 1.36) | 0.362 | 1.10 (0.89 to 1.36) | 0.366 | 1.10 (0.89 to 1.36) | 0.368 |
| No fixed abode recorded | 0.96 (0.93 to 1) | 0.040 | 0.96 (0.93 to 1.00) | 0.041 | 0.96 (0.93 to 1.00) | 0.04 | 0.96 (0.93 to 1.00) | 0.039 |
| Other | 0.92 (0.87 to 0.97) | 0.003 | 0.92 (0.87 to 0.97) | 0.003 | 0.92 (0.87 to 0.97) | 0.003 | 0.92 (0.87 to 0.97) | 0.003 |
| **Presented between 9am and 5pm** | 1 (0.99 to 1.01) | 0.754 | 1.00 (0.99 to 1.01) | 0.722 | 1.00 (0.99 to 1.01) | 0.748 | 1.00 (0.99 to 1.01) | 0.766 |
| **History of self-harm in previous 12 months** | 0.99 (0.97 to 1.01) | 0.353 | 0.99 (0.97 to 1.01) | 0.353 | 0.99 (0.97 to 1.01) | 0.353 | 0.99 (0.97 to 1.01) | 0.353 |
| **Brought by ambulance or emergency services** | 1.12 (1.09 to 1.14) | <0.001 | 1.12 (1.10 to 1.14) | <0.001 | 1.12 (1.10 to 1.14) | <0.001 | 1.12 (1.09 to 1.14) | <0.001 |
| **Received psychosocial assessment** |  |  |  |  |  |  |  |  |
| Yes | 1.08 (1.02 to 1.14) | 0.007 | 1.08 (1.02 to 1.14) | 0.008 | 1.08 (1.02 to 1.14) | 0.007 | 1.08 (1.02 to 1.14) | 0.007 |
| Uknown | 0.59 (0.54 to 0.64) | <0.001 | 0.59 (0.54 to 0.65) | <0.001 | 0.59 (0.54 to 0.64) | <0.001 | 0.59 (0.54 to 0.64) | <0.001 |
| **Admitted to ED medical assessment unit** |  |  |  |  |  |  |  |  |
| Yes | 1.78 (1.63 to 1.95) | <0.001 | 1.79 (1.64 to 1.96) | <0.001 | 1.78 (1.63 to 1.95) | <0.001 | 1.78 (1.63 to 1.95) | <0.001 |
| Uknown | 0.86 (0.78 to 0.94) | 0.001 | 0.86 (0.79 to 0.95) | 0.002 | 0.86 (0.78 to 0.94) | 0.001 | 0.86 (0.78 to 0.94) | 0.001 |
| **Admission status**  (ref, discharged from ED) |  |  |  |  |  |  |  |  |
| Medical admission | 2.88 (2.55 to 3.26) | <0.001 | 2.88 (2.55 to 3.26) | <0.001 | 2.88 (2.55 to 3.26) | <0.001 | 2.88 (2.55 to 3.26) | <0.001 |
| Psychiatric admission | 3.02 (2.72 to 3.36) | <0.001 | 2.04 (1.83 to 2.27) | <0.001 | 2.78 (2.50 to 3.09) | <0.001 | 3.65 (3.29 to 4.06) | <0.001 |
| Self-discharged | 0.61 (0.58 to 0.65) | <0.001 | 0.61 (0.58 to 0.65) | <0.001 | 0.61 (0.58 to 0.65) | <0.001 | 0.61 (0.58 to 0.65) | <0.001 |
| **Hospital type** (ref, general) |  |  |  |  |  |  |  |  |
| Children’s hospital | 1.00 (0.84 to 1.19) | 0.997 | 1.00 (0.84 to 1.20) | 0.999 | 1.00 (0.84 to 1.19) | 0.998 | 1.00 (0.84 to 1.19) | 0.995 |
| Local injury unit | 0.93 (0.90 to 0.96) | <0.001 | 0.93 (0.90 to 0.96) | <0.001 | 0.93 (0.90 to 0.96) | <0.001 | 0.93 (0.90 to 0.96) | <0.001 |
| Tertiary | 0.97 (0.89 to 1.05) | 0.438 | 0.97 (0.89 to 1.06) | 0.475 | 0.97 (0.89 to 1.05) | 0.445 | 0.97 (0.89 to 1.05) | 0.425 |
| **Psychiatric in-patient facilities onsite** | 1.02 (0.95 to 1.08) | 0.638 | 1.01 (0.95 to 1.08) | 0.685 | 1.01 (0.95 to 1.08) | 0.646 | 1.02 (0.95 to 1.08) | 0.622 |
| **Dedicated liaison psychiatry services onsite** | 1.03 (0.99 to 1.08) | 0.118 | 1.03 (0.99 to 1.08) | 0.112 | 1.03 (0.99 to 1.08) | 0.117 | 1.03 (0.99 to 1.08) | 0.121 |
| **Hospital location**  (ref, other city) |  |  |  |  |  |  |  |  |
| Dublin city | 0.98 (0.91 to 1.05) | 0.549 | 0.98 (0.91 to 1.05) | 0.53 | 0.98 (0.91 to 1.05) | 0.545 | 0.98 (0.91 to 1.05) | 0.555 |
| Town | 0.98 (0.87 to 1.1) | 0.672 | 0.98 (0.87 to 1.10) | 0.684 | 0.98 (0.87 to 1.10) | 0.674 | 0.97 (0.87 to 1.09) | 0.667 |
| **Constant** | 790.43 (685.61 to 911.27) | <0.001 | 785.80 (682.69 to 904.49) | <0.001 | 789.58 (685.08 to 910.01) | <0.001 | 792.10 (686.64 to 913.76) | <0.001 |

All models are adjusted general linear regression models using a gamma distribution and log link function. IDO=intentional drug overdose. ED=emergency department.

**Supplementary Table 6. Associations between cost and demographic, clinical and health service characteristics for presentations that resulted in psychiatric admission (Model 3; n=2,711) assuming length of stay in psychiatric setting of 7.3 days (mean), 4.5 days (estimated 25^th^ percentile), 6.1 days (estimated median), and 9.1 days (estimated 75^th^ percentile).**

|  | **LOS: 7.3 days** | | **LOS: 4.5 days** | | **LOS: 6.1 days** | | **LOS: 9.1 days** | |  |
| --- | --- | --- | --- | --- | --- | --- | --- | --- | --- |
|  | *b* (95% CI); p value | | *b* (95% CI); p value | | *b* (95% CI); p value | | *b* (95% CI); p value | |  |
| **Female** | 1.01 (1 to 1.02) | 0.062 | 1.01 (1.00-1.02) | 0.058 | 1.01 (1.00-1.02) | 0.061 | 1.01 (1.00-1.01) | 0.064 |  |
| **Age group**  (ref, 15-44 years) |  |  |  |  |  |  |  |  |  |
| <15 years | 0.98 (0.95 to 1) | 0.078 | 0.96 (0.93-1.00) | 0.073 | 0.97 (0.95-1.00) | 0.077 | 0.98 (0.96-1.00) | 0.079 |  |
| 45-64 years | 1.02 (1.01 to 1.04) | 0.002 | 1.03 (1.01-1.04) | 0.002 | 1.02 (1.01-1.04) | 0.002 | 1.02 (1.01-1.03) | 0.002 |  |
| 65+ years | 1.01 (0.97 to 1.05) | 0.564 | 1.02 (0.96-1.07) | 0.56 | 1.01 (0.97-1.05) | 0.563 | 1.01 (0.98-1.04) | 0.566 |  |
| **IDO (X60-X64)** | 1.01 (0.99 to 1.02) | 0.456 | 1.01 (0.99-1.03) | 0.462 | 1.01 (0.99-1.02) | 0.457 | 1.00 (0.99-1.02) | 0.454 |  |
| **Attempted hanging (X70)** | 0.97 (0.95 to 0.98) | <0.001 | 0.95 (0.94-0.97) | <0.001 | 0.97 (0.95-0.98) | <0.001 | 0.97 (0.96-0.99) | <0.001 |  |
| **Firearm (X73)** | 0.98 (0.93 to 1.03) | 0.418 | 0.97 (0.90-1.04) | 0.417 | 0.98 (0.92-1.03) | 0.418 | 0.98 (0.94-1.03) | 0.418 |  |
| **Jumping from a height (X81)** | 0.96 (0.94 to 0.98) | 0.001 | 0.94 (0.91-0.97) | 0.001 | 0.96 (0.93-0.98) | 0.001 | 0.97 (0.95-0.99) | 0.001 |  |
| **Jumping or lying in front of moving object (X81)** | 0.95 (0.93 to 0.98) | <0.001 | 0.93 (0.90-0.97) | <0.001 | 0.95 (0.93-0.98) | <0.001 | 0.96 (0.94-0.98) | <0.001 |  |
| **Crashing motor vehicle (X82)** | 0.95 (0.93 to 0.97) | <0.001 | 0.93 (0.90-0.96) | <0.001 | 0.94 (0.92-0.97) | <0.001 | 0.96 (0.94-0.98) | <0.001 |  |
| **Self-immolation (X76)** | 0.96 (0.94 to 0.98) | <0.001 | 0.94 (0.92-0.97) | <0.001 | 0.96 (0.94-0.98) | <0.001 | 0.97 (0.95-0.98) | <0.001 |  |
| **Blunt object (X79)** | 0.96 (0.94 to 0.98) | <0.001 | 0.94 (0.91-0.97) | <0.001 | 0.96 (0.93-0.98) | <0.001 | 0.97 (0.95-0.98) | <0.001 |  |
| **Residence status**  (ref, household resident) |  |  |  |  |  |  |  |  |  |
| Hospital in-patient | 1.01 (0.97 to 1.06) | 0.567 | 1.02 (0.95-1.09) | 0.575 | 1.02 (0.96-1.07) | 0.569 | 1.01 (0.97-1.05) | 0.564 |  |
| Prisoner | - |  | - |  |  |  |  |  |  |
| No fixed abode recorded | 1.00 (0.97 to 1.02) | 0.714 | 0.99 (0.96-1.03) | 0.717 | 1.00 (0.97-1.02) | 0.714 | 1.00 (0.98-1.02) | 0.713 |  |
| Other | 0.96 (0.94 to 0.98) | 0.001 | 0.94 (0.91-0.98) | 0.001 | 0.96 (0.93-0.98) | 0.001 | 0.97 (0.95-0.99) | 0.001 |  |
| **Presented between 9am and 5pm** | 0.99 (0.98 to 1) | 0.068 | 0.99 (0.97-1.00) | 0.067 | 0.99 (0.98-1.00) | 0.067 | 0.99 (0.98-1.00) | 0.068 |  |
| **Presented at the weekend** | 0.99 (0.99 to 1) | 0.003 | 0.99 (0.98-1.00) | 0.004 | 0.99 (0.98-1.00) | 0.003 | 0.99 (0.99-1.00) | 0.003 |  |
| **Brought by ambulance or emergency services** | 1.05 (1.04 to 1.06) | <0.001 | 1.08 (1.06-1.09) | <0.001 | 1.06 (1.04-1.07) | <0.001 | 1.04 (1.03-1.05) | <0.001 |  |
| **Received psychosocial assessment** |  |  |  |  |  |  |  |  |  |
| Yes | 1.01 (0.96 to 1.06) | 0.809 | 1.01 (0.94-1.08) | 0.8 | 1.01 (0.95-1.06) | 0.807 | 1.00 (0.97-1.05) | 0.812 |  |
| Uknown | 0.95 (0.89 to 1.02) | 0.172 | 0.93 (0.96-1.05) | 0.175 | 0.95 (0.88-1.02) | 0.173 | 0.96 (0.91-1.02) | 0.171 |  |
| **Admitted to ED medical assessment unit** |  |  |  |  |  |  |  |  |  |
| Yes | 1.06 (1.02 to 1.1) | 0.001 | 1.09 (1.04-1.14) | 0.001 | 1.06 (1.03-1.10) | 0.001 | 1.05 (1.02-1.08) | 0.001 |  |
| Uknown | 1 (0.97 to 1.04) | 0.809 | 1.01 (0.96-1.09) | 0.813 | 1.00 (0.97-1.04) | 0.81 | 1.00 (0.98-1.03) | 0.808 |  |
| **Hospital type** (ref, general) |  |  |  |  |  |  |  |  |  |
| Children’s hospital | 0.94 (0.89 to 1) | 0.062 | 0.92 (0.84-1.00) | 0.054 | 0.94 (0.88-1.00) | 0.06 | 0.95 (0.91-1.00) | 0.064 |  |
| Local injury unit | 0.96 (0.89 to 1.02) | 0.203 | 0.94 (0.84-1.04) | 0.205 | 0.95 (0.88-1.03) | 0.204 | 0.96 (0.91-1.02) | 0.203 |  |
| Tertiary | 1.02 (0.97 to 1.06) | 0.502 | 1.02 (0.96-1.09) | 0.506 | 1.02 (0.97-1.07) | 0.503 | 1.01 (0.98-1.05) | 0.501 |  |
| **Constant** | 3648.07 (3472.42 to 3832.62) | <0.001 | 2383.05 (2216.91-2561.65) | <0.001 | 3331. 61 (3157-76-3517.76) | <0.001 | 4462.20 (4282.94-4648.96) | <0.001 |  |
| All models are adjusted general linear regression models using a gamma distribution and log link function. IDO=intentional drug overdose. ED=emergency department. | | | | | | | | | |

**Supplementary Table 7. Pregibon goodness of link tests for model 1 (total cost).**

| Model | Log-likelihood | Bayesian Information Criterion (BIC) | Akaike information criterion (AIC) |
| --- | --- | --- | --- |
| Log link | -218882.49 | 6.40 | 17.60 |
| Identity link | -219546.94 | 6.75 | 17.65 |


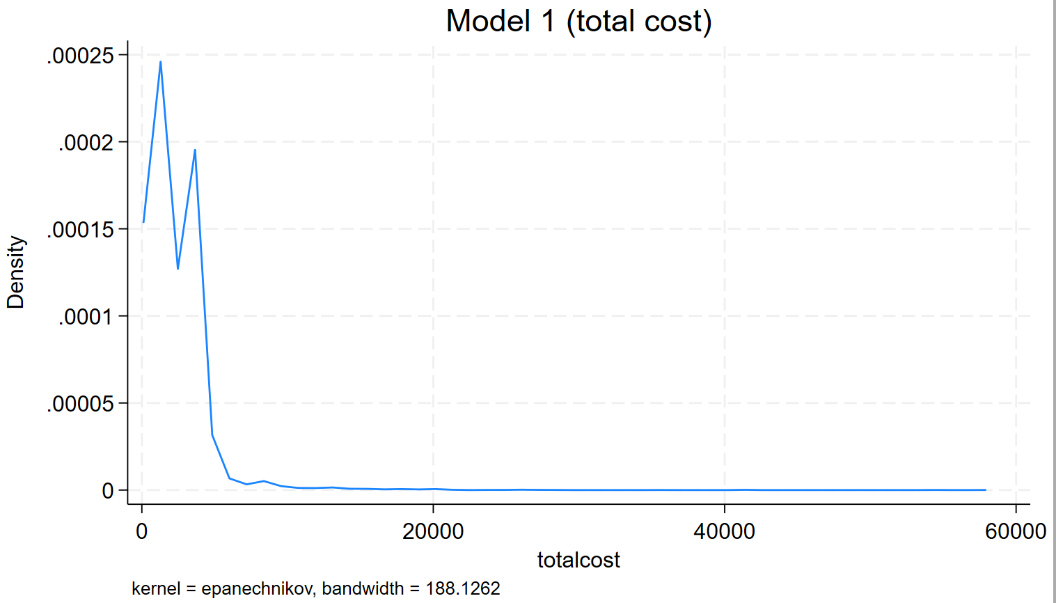
**
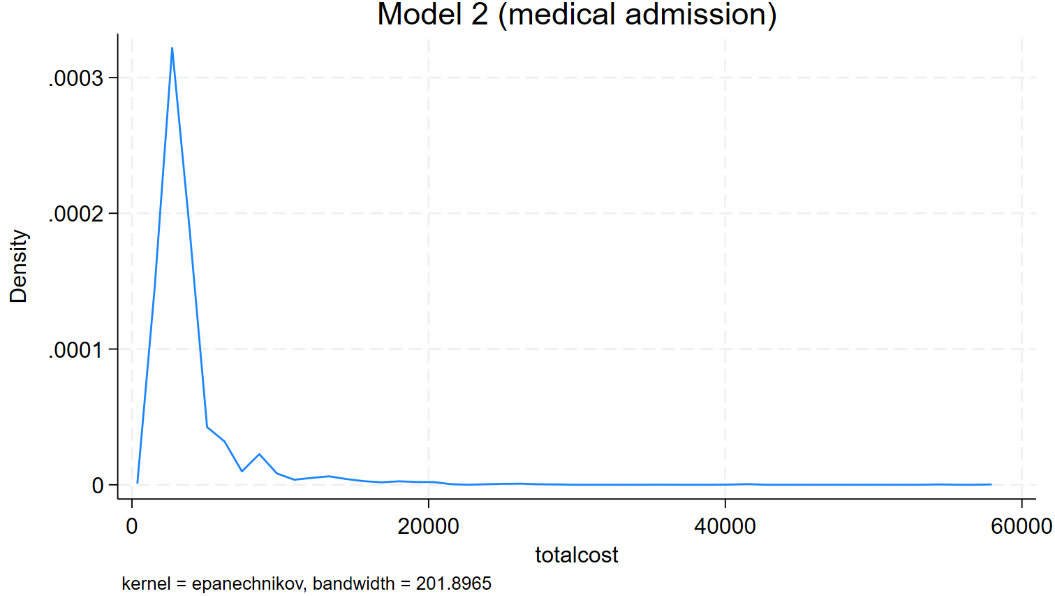

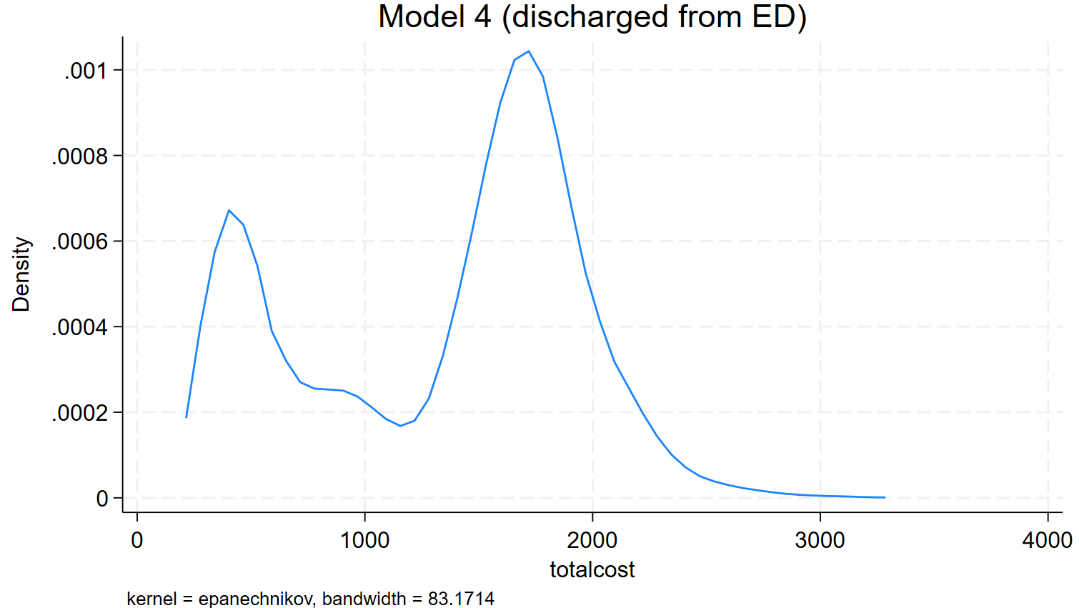

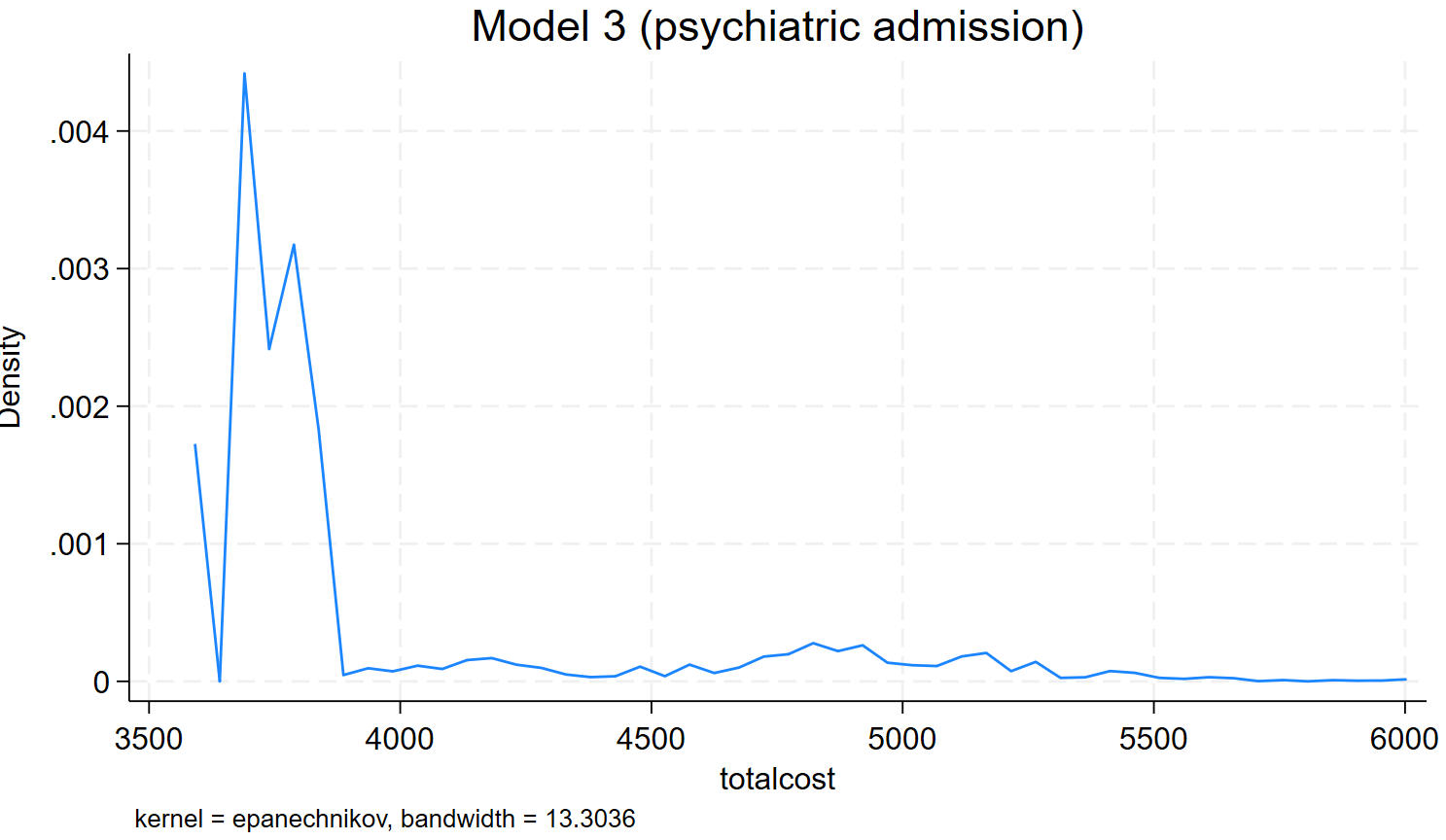
**

**Supplementary Figure 1. Distribution of cost via Kernel Density Estimation for total cost, medical admission, psychiatric admission and discharge from ED.**

**
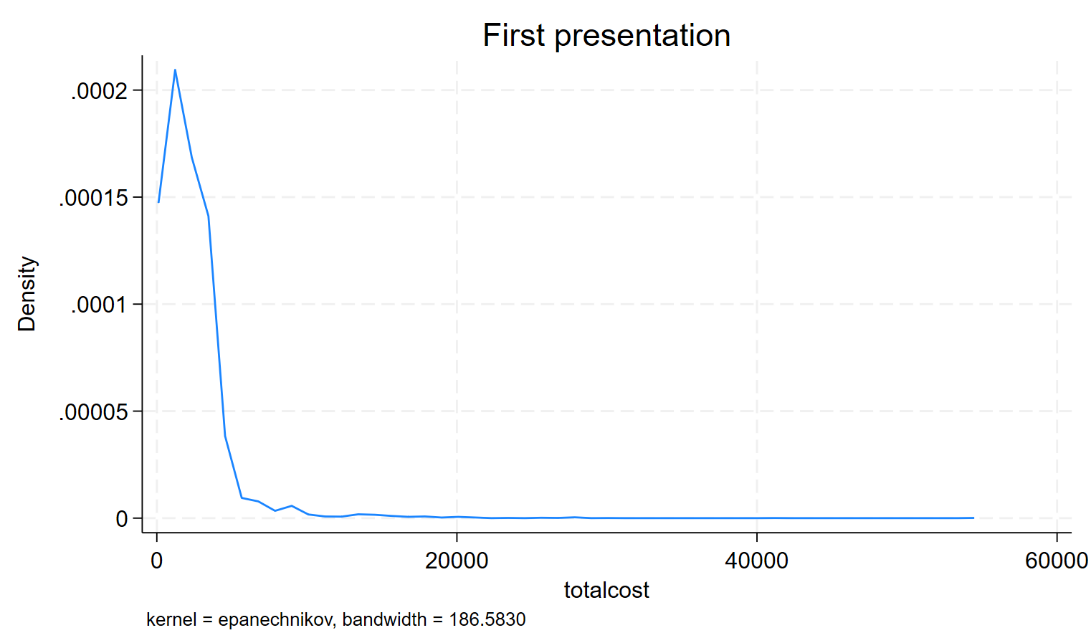

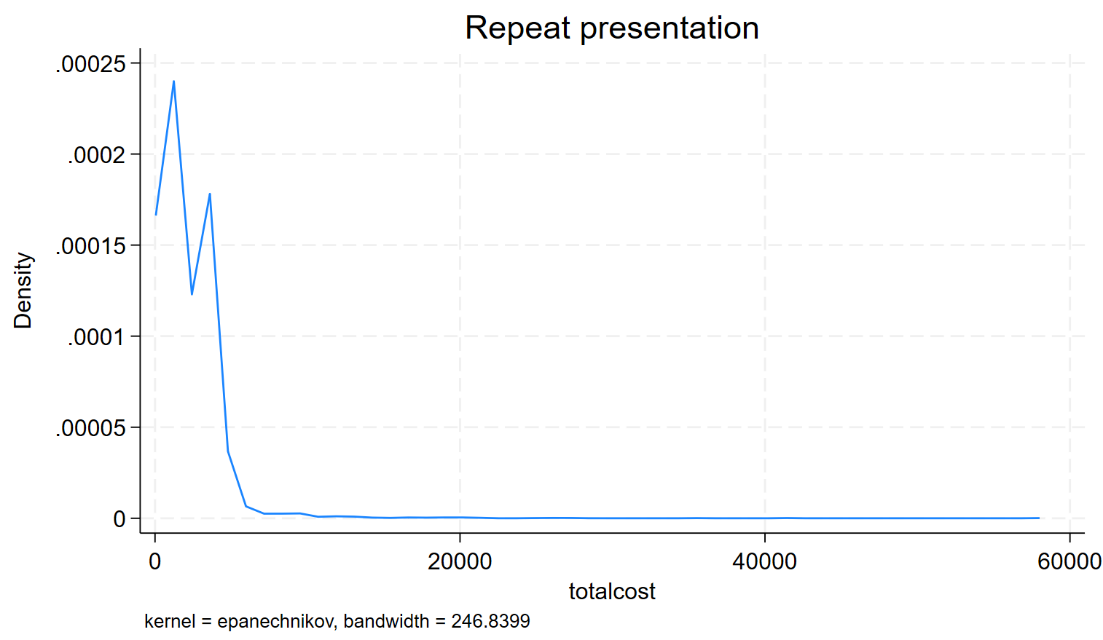
**

**Supplementary Figure 2. Distribution of cost via Kernel Density Estimation for first and repeat presentations.**

**References**

1. Pitman A, Tsiachristas A, Casey D, Geulayov G, Brand F, Bale E, et al. Comparing short-term risk of repeat self-harm after psychosocial assessment of patients who self-harm by psychiatrists or psychiatric nurses in a general hospital: Cohort study. Journal of Affective Disorders. 2020 Jul 1;272:158–65.

2. Department of Health. Department of health consolidated salary scales. Dublin: Department of Health; 2021 Jul.

3. HIQA. Guidelines for the Economic Evaluation of Health Technologies in Ireland 2020 [Internet]. Dublin: Health Information and Quality Authority.; [cited 2023 May 4]. Available from: https://www.hiqa.ie/reports-and-publications/health-technology-assessment/guidelines-economic-evaluation-health

4. Organization WH. ICD-10 : international statistical classification of diseases and related health problems : tenth revision [Internet]. World Health Organization; 2004 [cited 2021 Apr 29]. Available from: https://apps.who.int/iris/handle/10665/42980

5. A cost-effectiveness analysis of dialectical behaviour therapy for treating individuals with borderline personality disorder in the community | Request PDF. ResearchGate [Internet]. 2024 Dec 10 [cited 2025 Jan 28]; Available from: https://www.researchgate.net/publication/335588309_A_cost-effectiveness_analysis_of_dialectical_behaviour_therapy_for_treating_individuals_with_borderline_personality_disorder_in_the_community

6. Keegan C, Brick A, Bergin A, Wren MA, Henry E, Whyte R. Projections of expenditure for public hospitals in Ireland, 2018–2035, based on the Hippocrates Model [Internet]. ESRI; 2020 Dec [cited 2025 Jan 28]. Available from: https://esri.ie/publications/projections-of-expenditure-for-public-hospitals-in-ireland-2018-2035-based-on-the

7. HPO [Internet]. 2023 [cited 2025 Jan 28]. HIPE_Annual_Report_Latest. Available from: https://hpowp.com/abf/hipe_annual_report_latest/

8. Quinlivan L, Cooper J, Meehan D, Longson D, Potokar J, Hulme T, et al. Predictive accuracy of risk scales following self-harm: multicentre, prospective cohort study. Br J Psychiatry. 2017 Jun;210(6):429–36.

9. Smith S, Jiang J, Normand C, O’Neill C. Unit costs for non-acute care in Ireland 2016-2019. HRB Open Res. 2021;4:39.

10. Sadath A, Troya MI, Nicholson S, Cully G, Leahy D, Ramos Costa AP, et al. Physical and mental illness comorbidity among individuals with frequent self-harm episodes: A mixed-methods study. Frontiers in Psychiatry [Internet]. 2023 [cited 2023 May 4];14. Available from: https://www.frontiersin.org/articles/10.3389/fpsyt.2023.1121313

11. Cully G, Corcoran P, Leahy D, Cassidy E, Steeg S, Griffin E, et al. Factors associated with psychiatric admission and subsequent self-harm repetition: a cohort study of high-risk hospital-presenting self-harm. J Ment Health. 2021 Dec;30(6):751–9.

12. National Clinical Programme for Self Harm and Suicide Related Ideation – Implementation Advisory Group. National clinical programme for self-harm and suicide-related ideation: updating the national clinical programme for the assessment and management of patients presenting to the emergency department following self-harm. Dublin: Health Service Executive; 2022.

13. Department of Health. Department of health consolidated salary scales. Dublin: Department of Health; 2021 Jul.

14. HIQA. Guidelines for the Economic Evaluation of Health Technologies in Ireland 2020 [Internet]. Dublin: Health Information and Quality Authority.; [cited 2023 May 4]. Available from: https://www.hiqa.ie/reports-and-publications/health-technology-assessment/guidelines-economic-evaluation-health

15. Unit Costs of Health and Social Care 2021 | PSSRU [Internet]. [cited 2025 Jan 28]. Available from: https://www.pssru.ac.uk/project-pages/unit-costs/unit-costs-of-health-and-social-care-2021/
